# Flavour-enhanced cortisol release during gum chewing

**DOI:** 10.1371/journal.pone.0173475

**Published:** 2017-04-05

**Authors:** Yoko Hasegawa, Yoshihisa Tachibana, Takahiro Ono, Hiromitsu Kishimoto

**Affiliations:** 1 Department of Dentistry and Oral Surgery, Hyogo College of Medicine, Nishinomiya, Hyogo, Japan; 2 Division of System Neuroscience, Kobe University Graduate School of Medicine, Kobe, Japan; 3 Division of Comprehensive Prosthodontics, Niigata University Graduate School of Medical and Dental Sciences, Niigata, Japan; University of Michigan, UNITED STATES

## Abstract

There is some evidence to suggest that chewing gum reduces chronic stress. However, it remains controversial how the taste and odour properties of chewing gum influence stress. The present study was designed to investigate this issue in human subjects. Using an enzyme-linked immunosorbent assay, we tested salivary cortisol concentration, which is thought to be a stress marker, in 96 adults who chewed gum with different combinations of taste and odour. Subjects could discriminate between the types of gum without prior information. Salivary cortisol concentrations were highest and lowest for the subjects who chewed the most flavourful gum and the least flavourful gum, respectively. These findings suggest that the salivary cortisol level during gum chewing is not a marker of negative emotions (i.e., stressful conditions) as traditionally considered but, rather, an index of positive emotions that can facilitate biological responses to overcome stressful conditions.

## Introduction

Chewing is an essential behaviour in daily life. In addition to its well-known function in digestion and absorption, chewing is tightly linked to hedonic (emotional) systems in the brain [1]. The hedonic aspect of chewing is greatly affected by the taste and odour of food [2]. Chewing palatable foods (e.g., a gum with good taste and odour) can induce positive emotions [3]. The underlying neural mechanism of this phenomenon begins with detection via taste and odorant chemoreceptors [2]. Subsequently, sensory signals are individually processed in the gustatory and olfactory neural circuits in the brain and integrated as a sensation of flavour in the insular and orbitofrontal cortical areas [4–6]. Cortical flavour information is further transmitted to the brain reward system, including the nucleus accumbens, midbrain dopamine areas, amygdala, and hypothalamus [6–8].

It is generally believed that chewing can relieve stressful conditions [9–11]. Stress is considered to represent the body’s response to physical and psychological threats (stressors) [12]. The stress response is associated with increased neuronal activity of the hypothalamic-pituitary-adrenal axis, which leads to the release of adrenal cortical hormone (cortisol) [13, 14]. Consequently, cortisol is a widely used biomarker of stress, and measurement of salivary cortisol levels is a well-established method to evaluate for physiological stress [15, 16]. Several physiological studies, however, have reported that cortisol levels are increased [17, 18] or remain unchanged by chewing [19]. In addition, it remains controversial whether the intake of flavourful foods can truly exert stress-relieving effects [20]. Thus, the present study was designed to re-examine the influence of taste and odour properties of chewing on stress. To address this issue, we measured cortisol release in the saliva of subjects who chewed gums with different combinations of taste and odour (including unpleasant flavour).

## Materials and methods

This study followed a randomized controlled cross-over design. Prior to the study, we obtained approval for the study from the Hyogo College of Medicine Ethics Committee (No.1318) on September 4, 2012. This research is registered in the University hospital Medical Information Network (UMIN) Clinical Trials Registry (UMIN000017039) after beginning enrolment of participants. Regarding the delay in registering this study, our study did not involve an investigation on pharmaceuticals or medical device use. In addition, the risk of the study was extremely low (and only included gum-chewing by the participants) [21]. Subjects who were judged to be appropriate for the experiment by a physician were recruited by the authors from September 4, 2012 through March 16, 2014. All subjects provided written informed consent. Inclusion criteria of the subjects were as follows: 1) a healthy dentulous volunteer who possessed the ability to chew our tested gums and 2) non-smoker status. The exclusion criterion was a person who would not agree to provide informed consent for our study. The subjects were instructed not to take any medication at the time of measurements. In all experiments, the subjects were asked to abstain from eating for more than 3 hours after breakfast so that they could perform the behavioural tests (chewing gums) before noon (approximately 11:30 AM). Throughout the study, the subjects sat on a chair in a quiet room with an ambient temperature of 25°C.

### Tested gums

We prepared several types of gum with different combinations of taste and odour: (1) no taste/no odour gum (C-gum), (2) no taste/lemon odour gum (O-gum), (3) sweet taste/no odour gum (T-gum), and (4) sweet taste/lemon odour gum (TO-gum). Gum were manufactured by Lotte Company and adjusted to have the same hardness at approximately 1 minute after the subjects began chewing. The speed of chewing and the chewing side was not restricted in Experiments I and II. In Experiment III, we used Salmiakki Chewing Gum (SL-gum; Fazer, Finland) as the unpalatable gum. Petrowski et al. [22] have reported that the speed of chewing is associated with subjects’ psychosocial stress. Thus, in Experiment III, we instructed the subjects to chew the tested gum with a comfortable rhythm at a constant speed of 70 times/min using a metronome sound [23].

### Treatment of saliva samples

**Fig 1:**
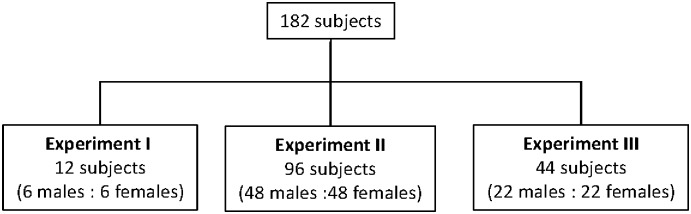
CONSORT flow diagram.

Saliva samples from the subjects were weighed (total saliva weight) and centrifuged (4000 rpm, 5 min). Following this, 300 μl of the supernatant was transferred into three Eppendorf tubes in 100-μl aliquots each, and samples were weighed to calculate the mass of the saliva and then cryopreserved. The mass was used to estimate total saliva volume (ml). The cryopreserved saliva was then used to measure the salivary cortisol concentration using a Cortisol Parameter^™^ Assay Kit (R&D Systems, Minneapolis, MN) and a UV/VIS (ultraviolet/visible) microplate spectrophotometer (SPECTRAmax PLUS384; Molecular Devices Corp., Tokyo, Japan). The derivation of the analytic sample is summarized in Fig 1.

### Experiment I: Temporal change in cortisol concentration during gum chewing

Twelve healthy adults (six males and six females, 24.6 ± 1.8 years old, mean ± standard deviation) were selected from the staff and students of Hyogo College of Medicine. We first examined the validity of our saliva collection method using 50-ml centrifuge tubes (Iwaki, Tokyo, Japan) by comparing it with the conventional method using Salivette (Sarstedt K.K., Tokyo, Japan) (see Discussion). Saliva sampled for 1 minute in a resting state by the 50-ml centrifuge tube method was designated as REST, while saliva sampled by the Salivette method was designated as REST2. The REST and REST2 were sampled in a random order.

Following verification that the 50-ml tube method was identical to the Salivette method, we tested moment-by-moment changes in salivary cortisol concentration during gum chewing. We collected the saliva for 1 minute at different times: before (rest), during, and after (just after, 10, 20, 30, and 60 min after) gum chewing. In this protocol, we sampled the saliva using the Salivette method (except for during gum chewing sampled by the 50-ml tube method; see Discussion). Throughout Experiment I, we used TO-gum as the tested gum.

### Experiment II: Influence of taste and odour on cortisol release during gum chewing

Ninety-six healthy adults (48 males and 48 females, 24.7 ± 3.3 years old) participated in this experiment. To examine the influence of gum taste and odour on cortisol release, we prepared several types of gum with different tastes and odours (see above). The subjects chewed the gum in a random order (by Latin square design), and saliva was collected using the 50-ml tube method after each minute of chewing. The subjects rated the taste and odour of each tested gum using the visual analogue scale (VAS). Then they rinsed their mouth with mineral water, and rested for 5 minutes before changing the tested gum.

### Experiment III: Comparison of cortisol release between palatable and unpalatable gums

Forty-four healthy adults (22 males and 22 females, 27.9 ± 8.1 years old) participated in this experiment. To test whether emotional states during gum chewing can influence cortisol release, the subjects were asked to chew a palatable (TO-gum, see above) and (Salmiakki Chewing Gum, Fazer, Finland; SL-gum) gum; the latter is not preferred by most Japanese subjects. The experimental procedure was the same as in Experiment II.

### Statistical analysis

The population sample size was estimated using a preliminary analysis of the data to avoid incorrect inferences in the interpretation of results (typically, α = 0.05). In Experiment I, we set a power level of 0.8 and effect size of 0.8 for detecting a mean change using a paired *t*-test with α = 0.05. In experiments II and III, to obtain more reliable data, we set a power level of 0.95 and effect size of 0.5 for using paired *t*-tests with α = 0.004 (= 0.05/12). Data were assessed for normality and homoscedasticity. To test the relationship between REST and REST2 conditions, we calculated the intraclass correlation coefficient (ICC). For the data where the normality and homoscedasticity were confirmed, we used a paired *t*-test to compare the tested two groups. When normality and homoscedasticity were not confirmed, we used a Wilcoxon signed-rank test. To compare the values among multiple groups, we performed repeated-measures of ANOVA (analysis of variance) or Friedman tests were performed for the data where the normality and homoscedasticity were confirmed or denied, respectively. Multiple comparison tests were performed by using paired *t*-tests or Wilcoxon signed-rank tests depending on the normality and homoscedasticity, with P<0.05 as a statistically significant difference. The multiplicity was corrected by Bonferroni’s methods. All statistical analyses were performed using SPSS statistics version 22.0 software (IBM, Chicago, IL).

## Results

### Experiment I. Salivary cortisol was influenced by chewing behaviour

We first verified the validity of our simple salivary cortisol concentration measurement in a 50-ml centrifuge tube by comparing it to the conventional Salivette method. The mean cortisol concentration measured by the centrifuge tube (REST) was 1.40 ng/mL (0.70–3.15, confidence interval), while the concentration measured by the Salivette (REST2) was 1.55 ng/mL (1.05–3.15, confidence interval). We calculated the ICC between the two methods and found the ICC to be 0.72 (confidence interval; 0.46–0.87, p<0.001). We also estimated the relationship between the cortisol concentration measured by the centrifuge tube and that measured by the Salivette as follows:

Cortisol concentration (Salivette) = Cortisol concentration (centrifuge tube)/0.935.

Thus, we verified that our Salivette method was the appropriate one to measure the cortisol concentration.

**Fig 2:**
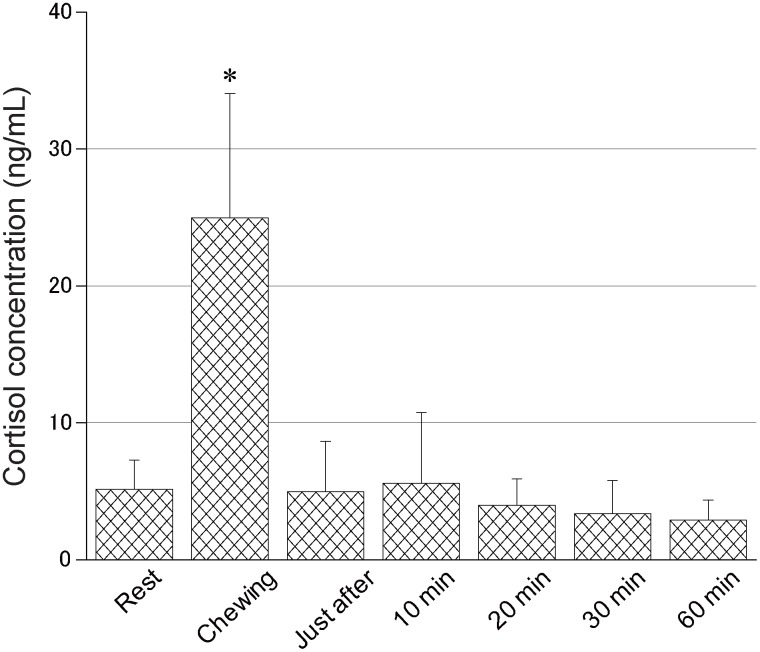
Time course of chewing-associated cortisol release. The salivary cortisol concentration was measured at seven different stages (N = 12), which are described as follows: rest (before chewing), chewing (during chewing), immediately after chewing, and 10, 20, 30, and 60 min after chewing of taste and odour gum (TO-gum). Error bars indicate the 95% confidence interval. There was a significant difference in salivary cortisol concentrations at rest and those during gum chewing. *: vs. rest (*P* < 0.008 [0.05/6]), according to Friedman tests followed by Bonferroni’s correction for multiple comparisons.

We next examined the temporal change in salivary cortisol concentration induced by chewing TO-gum (Fig 2). During gum chewing, there was a marked increase in cortisol concentration when compared with the resting condition (*P* < 0.001, using Friedman tests followed by Bonferroni’s correction for multiple comparisons). This increase was transient, with the cortisol concentration returning to control levels immediately after the gum chewing. These data indicated that the salivary cortisol concentration was strongly influenced by chewing behaviour.

### Experiment II. Odour and taste modulated cortisol release during gum chewing

**Fig 3:**
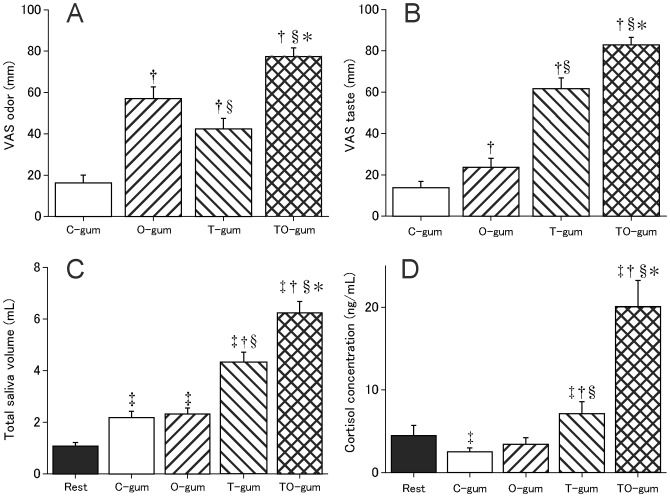
Flavour modulation of cortisol release during gum chewing. A: The visual analogue scale of the tested gum odour component B: The visual analogue scale of the taste component C: Total saliva volume D: Cortisol concentration. Significant differences are represented by individual symbols. *: vs. T-gum; §: vs. O-gum; †: vs. C-gum; ‡: vs. Rest. Statistical significance was *P* < 0.013 (0.05/4) or 0.01 (0.05/5), according to Friedman tests followed by Bonferroni’s correction for multiple comparisons.

In this experiment, we tested whether the taste and odour of gum could influence cortisol release in the saliva during chewing behaviour. Prior to the cortisol measurement, we compared the subjective scores of odour and taste of the tested gums (see Materials and methods) using the VAS (Fig 3). The TO- and C-gums were rated highest and lowest, respectively, for both odour and taste (Fig 3A and 3B). The O- and T-gums had intermediate scores. These data suggested that subjects are able to discriminate between the types of gum without prior information.

We next examined total saliva volume while the subjects chewed the four types of gum. The TO-gum generated the largest saliva volume, with the next largest volume found while chewing the T-gum (Fig 3C). The saliva volumes while chewing the C- and O-gums were larger than those in the resting condition (Rest). We then tested the salivary cortisol concentration and found that the TO-gum generated the highest concentration, followed by the T-gum (Fig 3D). Interestingly, the cortisol concentration generated by the C-gum was lower than that in the Rest condition. These findings suggested that salivary cortisol did not simply reflect the sensorimotor response of chewing behaviour but might reflect the cognitive/motivational aspects of chewing.

### Experiment III. Motivational modulation of cortisol release during gum chewing

**Fig 4:**
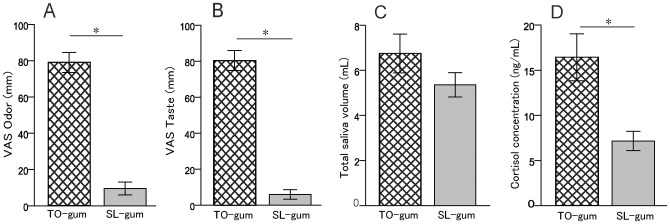
The motivational bias of cortisol release during gum chewing. The subjects were asked to chew a palatable (TO-gum, positive motivational outcome) and unpalatable (SL-gum, negative motivational outcome) gum. A: The visual analogue scale of odour B: The visual analogue scale of taste C: Total saliva volume D: Cortisol concentration Statistical significance was *P* <0.05, according to Wilcoxon signed-rank tests.

Finally, we examined whether internal motivational state could affect cortisol release by measuring salivary cortisol while the subjects chewed palatable TO- and unpalatable SL-gums. The odour (Fig 4A) and taste (Fig 4B) VAS indicated that the TO-gum was preferred when compared with the SL-gum. Although the total saliva volume was similar between the TO- and SL-gums (Fig 4C), the cortisol concentration in the TO-gum group was significantly higher than that in the SL-gum group. These findings indicated that cortisol concentration was strongly modulated by internal motivational state.

## Discussion

We found that the most flavourful gum induced the largest release of salivary cortisol, while the least flavourful gum induced the smallest release. Further examination revealed that the emotional state (positive vs. negative) of subjects was an important factor influencing the amount of cortisol released during gum chewing.

### Methodological considerations

Stress is a widely accepted critical factor in the incidence of lifestyle-related diseases, such as cerebrovascular and ischemic heart disease [24, 25]. Plasma cortisol, which is associated with the body’s response to stressors, is a well-recognized marker of stress [26]. In response to stressors, such as dehydration, heat shock, or viral infection, corticotropin-releasing hormone (CRH) is secreted from the hypothalamus, which facilitates the secretion of adrenocorticotropic hormone (ACTH) in the pituitary. The action of ACTH, in turn, increases cortisol release from the adrenal cortex [13, 14]. Thus, cortisol is a good marker of stress [26]. However, accurately measuring plasma cortisol is challenging, because blood collection is stressful and can affect the concentration of endogenous cortisol. On the other hand, salivary cortisol is positively correlated with plasma cortisol; therefore, it can be used as a non-invasive measure of stress [27–29]. Previous studies have reported that the salivary cortisol concentration is not influenced by gum chewing [19]. The initial aims of this study were to investigate whether gum chewing affects cortisol levels and the modulation of cortisol release by the taste and odour properties of the tested gum.

In Experiment I, we conducted a comparative study regarding the validity of different salivary cortisol collection methods. The most popular method is the Salivette method using a cotton roll [28, 30]. However, it is difficult to retain the cotton in the oral cavity during gum chewing. Therefore, we used a centrifuge tube to collect the saliva stored in the oral cavity while subjects were chewing gum without cotton. Our data showed that the salivary cortisol measured by the centrifuge tube (during the resting state) was comparable to the cortisol measured by the Salivette method. In addition, the cortisol concentration in stimulated saliva by gum chewing was not influenced by the amount of saliva [30]. Thus, we adopted this simple centrifuge tube method for the subsequent experiments.

### Gum chewing increases the salivary cortisol level

Chewing of TO-gum transiently increased the release of cortisol when compared with resting conditions (Fig 2); the cortisol concentration returned to control levels immediately after the subjects finished chewing. This suggested that the concentration of cortisol may dynamically reflect the sensorimotor response triggered by TO-gum chewing. Several studies have reported that chewing behaviour reduces the levels of stress hormones. Tahara et al. [11] and Scholey et al. [10] have found that chewing under the loading of mental stress reduces the level of salivary cortisol. An MRI study has demonstrated that gum chewing relieves stress by inhibiting the propagation of stress-related information in the brain. [31] These results are inconsistent with our results. On the other hand, our data are supported by the studies of Gray et al. [18] and Smith [17]; they reported that cortisol levels are increased by gum chewing. Smith [17] has reported that the chewing-induced elevation of cortisol levels reflects the increased arousal level and cognitive processing secondary to task facilitation, suggesting that, in our study, the cortisol elevation resulting from the arousal-attention process may override the cortisol downregulation due to the stress-relieving effect.

### Different taste and odour modulate the cortisol release during gum chewing

We then examined how flavour information (i.e., taste and odour) influenced cortisol release during gum chewing (Fig 3). Our VAS analysis confirmed that the TO- and C-gum were the most and least flavourful gums, respectively. A subsequent experiment showed that the TO-gum elicited the highest release of cortisol, while the lowest release occurred when subjects chewed the flavourless C-gum. Interestingly, the concentration of cortisol during C-gum or O-gum chewing was lower than under resting conditions. This indicated that cortisol concentration does not simply reflect motor output and/or somatosensory (tactile/proprioceptive) and olfactory information. Rather, the concentration of cortisol could be directly modulated by a hedonic component of the gums (i.e., the flavour). This interpretation contradicts the traditional concept that salivary cortisol levels increase in response to physical and psychological stressors; rather, the upregulation of cortisol response following flavorful-gum chewing may exert anti-stress effects on confronting/upcoming stressors such as fear and anxiety [17, 32].

### Motivational modulation of cortisol levels during gum chewing

We found that salivary cortisol levels were increased in response to flavourful gum and decreased or were unchanged in response to flavourless gum. Next, we sought to examine whether internal motivational biases could affect cortisol release during gum chewing. Further examination revealed that cortisol release during palatable TO-gum chewing was significantly higher than that during unpalatable SL-gum chewing (Fig 4). These data suggested that positive motivational outcomes triggered by the palatable gum activated reward systems in the brain, while negative motivational outcomes triggered by the unpalatable gum activated punishment systems in the brain. The good gustatory information processed in brain taste areas, such as the parabrachial nucleus and insular cortex, could activate brain reward systems, such as the nucleus accumbens and ventral tegmental area [33]. Conversely, bad gustatory information could activate brain punishment systems, such as the amygdala and lateral habenula [34]. Both reward and punishment brain areas strongly interact with the hypothalamus [35]; therefore, good and bad taste information could have opposing effects on neuronal activity in the hypothalamus [36] and differentially modulate the neuroendocrine systems in the hypothalamic-pituitary-adrenal axis that controls endogenous cortisol levels.

### Implications of the study

We demonstrated that salivary cortisol levels during gum chewing are positively associated with internal motivational levels. These data suggest that the concentration of salivary cortisol during gum chewing is not a marker of negative emotions (i.e., stressful conditions), as traditionally considered, but rather, an index of positive emotions that can facilitate biological responses to overcome stressful conditions.

## Supporting information

S1 Fig Ethics approval (original in Japanese). (PDF)

S2 Fig Ethics approval (translated in English). (PDF)

S1 Text Ethics Committee application form and implementation plan document (original in Japanese.) (PDF)

S2 Text Ethics Committee application form and implementation plan document (translated in English). (PDF)

S3 Text All data for experiment 1. (XLSX)

S4 Text All data for experiment 2. (XLSX)

S5 Text All data for experiment 3. (XLSX)

S6 Text CONSORT checklist. (DOC)

## References

[1] Deiss V, Rossignol L, Bourdiol P. Negative emotional state shortens the duration of the chewing sequence. Food quality and preference. 2009;20(1):57–61.

[2] Spielman AI. Chemosensory function and dysfunction. Crit Rev Oral Biol Med. 1998;9(3):267–91. 9715366 10.1177/10454411980090030201

[3] Carnell S, Kim Y, Pryor K. Fat brains, greedy genes, and parent power: a biobehavioural risk model of child and adult obesity. International review of psychiatry (Abingdon, England). 2012;24(3):189–99. 10.3109/09540261.2012.676988 22724640

[4] Kringelbach ML. The human orbitofrontal cortex: linking reward to hedonic experience. Nat Rev Neurosci. 2005;6(9):691–702. Epub 2005/09/02. 10.1038/nrn1747 16136173

[5] Buck L, Axel R. A novel multigene family may encode odorant receptors: a molecular basis for odor recognition. Cell. 1991;65(1):175–87. Epub 1991/04/05. 1840504 10.1016/0092-8674(91)90418-x

[6] Shepherd GM. Smell images and the flavour system in the human brain. Nature. 2006;444(7117):316–21. 10.1038/nature05405 17108956

[7] Haber SN, Knutson B. The reward circuit: linking primate anatomy and human imaging. Neuropsychopharmacology: official publication of the American College of Neuropsychopharmacology. 2010;35(1):4–26. 19812543 10.1038/npp.2009.129 PMC3055449

[8] Ventura R, Alcaro A, Puglisi-Allegra S. Prefrontal cortical norepinephrine release is critical for morphine-induced reward, reinstatement and dopamine release in the nucleus accumbens. Cerebral cortex (New York, NY: 1991). 2005;15(12):1877–86. Epub 2005/02/25. 10.1093/cercor/bhi066 15728739

[9] Smith A. Effects of chewing gum on cognitive function, mood and physiology in stressed and non-stressed volunteers. Nutritional Neuroscience. 2010;13(1):7–16. 10.1179/147683010X12611460763526 20132649

[10] Scholey A, Haskell C, Robertson B, Kennedy D, Milne A, Wetherell M. Chewing gum alleviates negative mood and reduces cortisol during acute laboratory psychological stress. Physiology & Behavior. 2009;97(3–4):304–12. 19268676 10.1016/j.physbeh.2009.02.028

[11] Tahara Y, Sakurai K, Ando T. Influence of chewing and clenching on salivary cortisol levels as an indicator of stress. Journal of prosthodontics: official journal of the American College of Prosthodontists. 2007;16(2):129–35. 17362423 10.1111/j.1532-849X.2007.00178.x

[12] Lovallo WR. Stress and health: Biological and psychological interactions: Sage publications; 2004.

[13] Kaufman J, Plotsky PM, Nemeroff CB, Charney DS. Effects of early adverse experiences on brain structure and function: Clinical implications. Biol Psychiatry. 2000;48(8):778–90. 11063974 10.1016/s0006-3223(00)00998-7

[14] Ladd CO, Huot RL, Thrivikraman KV, Nemeroff CB, Plotsky PM. Long-term adaptations in glucocorticoid receptor and mineralocorticoid receptor mRNA and negative feedback on the hypothalamo-pituitary-adrenal axis following neonatal maternal separation. 2004;55(4):367–75. 10.1016/j.biopsych.2003.10.007 14960289

[15] Dinan TG, Quigley EMM, Ahmed SMM, Scully P, O'Brien S, O'Mahony L, et al Hypothalamic-pituitary-gut axis dysregulation in irritable bowel syndrome: Plasma cytokines as a potential biomarker? Gastroenterology. 2006;130(2):304–11. 10.1053/j.gastro.2005.11.033 16472586

[16] Hellhammer DH, Wuest S, Kudielka BM. Salivary cortisol as a biomarker in stress research. Psychoneuroendocrinology. 2009;34(2):163–71. 10.1016/j.psyneuen.2008.10.026 19095358

[17] Smith A. Effects of chewing gum on cognitive function, mood and physiology in stressed and non-stressed volunteers. Nutritional Neuroscience. 2010;13(1):7–16. 10.1179/147683010X12611460763526 20132649

[18] Gray G, Miles C, Wilson N, Jenks R, Cox M, Johnson AJ. The contrasting physiological and subjective effects of chewing gum on social stress. Appetite. 2012;58(2):554–8. 10.1016/j.appet.2011.11.013 22123610

[19] Matsui F, Koh E, Yamamoto K, Sugimoto K, Sin H-S, Maeda Y, et al Liquid Chromatography-tandem Mass Spectrometry (LC-MS/MS) Assay for Simultaneous Measurement of Salivary Testosterone and Cortisol in Healthy Men for Utilization in the Diagnosis of Late-onset Hypogonadism in Males. Endocrine Journal. 2009;56(9):1083–93. 19734692 10.1507/endocrj.k09e-186

[20] Pecoraro N, Reyes F, Gomez F, Bhargava A, Dallman MF. Chronic stress promotes palatable feeding, which reduces signs of stress: Feedforward and feedback effects of chronic stress. Endocrinology. 2004;145(8):3754–62. 10.1210/en.2004-0305 15142987

[21] Ministry of Health, Labour and Welfare (Japan). Ethic guideline about clinical studies. 2008:1–25. http://www.mhlw.go.jp/general/seido/kousei/i-kenkyu/rinsyo/dl/shishin.pdf

[22] Petrowski K, Wintermann G-B, Joraschky P, Paessler S. Chewing after stress: Psychosocial stress influences chewing frequency, chewing efficacy, and appetite. Psychoneuroendocrinology. 2014;48:64–76. 10.1016/j.psyneuen.2014.06.008 24997349

[23] Hasegawa Y, Ono T, Sakagami J, Hori K, Maeda Y, Hamasaki T, et al Influence of voluntary control of masticatory side and rhythm on cerebral hemodynamics. Clin Oral Investig. 2009. Epub 2009/08/28. 10.1007/s00784-009-0338-5 19711107

[24] Manuck SB, Kaplan JR, Matthews KA. Behavioral antecedents of coronary heart disease and atherosclerosis. Arteriosclerosis. 1986;6(1):2–14. 3510615 10.1161/01.atv.6.1.2

[25] Iso H, Date C, Yamamoto A, Toyoshima H, Tanabe N, Kikuchi S, et al Perceived mental stress and mortality from cardiovascular disease among Japanese men and women: the Japan Collaborative Cohort Study for Evaluation of Cancer Risk Sponsored by Monbusho (JACC Study). Circulation. 2002;106(10):1229–36. Epub 2002/09/05. 12208798 10.1161/01.cir.0000028145.58654.41

[26] Kirschbaum C, Pirke KM, Hellhammer DH. The 'Trier Social Stress Test'—a tool for investigating psychobiological stress responses in a laboratory setting. Neuropsychobiology. 1993;28(1–2):76–81. Epub 1993/01/01. doi: 119004 8255414 10.1159/000119004

[27] Kirschbaum C, Hellhammer DH. Salivary cortisol in psychobiological research: an overview. Neuropsychobiology. 1989;22(3):150–69. Epub 1989/01/01. doi: 118611 2485862 10.1159/000118611

[28] Kirschbaum C, Hellhammer DH. Salivary cortisol in psychoneuroendocrine research: recent developments and applications. Psychoneuroendocrinology. 1994;19(4):313–33. Epub 1994/01/01. 8047637 10.1016/0306-4530(94)90013-2

[29] Kirschbaum C, Wolf OT, May M, Wippich W, Hellhammer DH. Stress- and treatment-induced elevations of cortisol levels associated with impaired declarative memory in healthy adults. Life Sci. 1996;58(17):1475–83. 8622574 10.1016/0024-3205(96)00118-x

[30] Groschl M, Rauh M. Influence of commercial collection devices for saliva on the reliability of salivary steroids analysis. Steroids. 2006;71(13–14):1097–100. 10.1016/j.steroids.2006.09.007 17070563

[31] Yu H, Chen X, Liu J, Zhou X. Gum chewing inhibits the sensory processing and the propagation of stress-related information in a brain network. PloS one. 2013;8(4):e57111 10.1371/journal.pone.0057111 23573184 PMC3616056

[32] Allen AP, Smith AP. A review of the evidence that chewing gum affects stress, alertness and cognition. Journal of Behavioral and Neuroscience Research. 2011;9(1):7–23.

[33] Yamamoto T. Neural substrates for the processing of cognitive and affective aspects of taste in the brain. Arch Histol Cytol. 2006;69(4):243–55. 17287579 10.1679/aohc.69.243

[34] Matsumoto M, Hikosaka O. Representation of negative motivational value in the primate lateral habenula. Nat Neurosci. 2009;12(1):77–84. 10.1038/nn.2233 19043410 PMC2737828

[35] Small DM, Zatorre RJ, Dagher A, Evans AC, Jones-Gotman M. Changes in brain activity related to eating chocolate—From pleasure to aversion. Brain. 2001;124:1720–33. 11522575 10.1093/brain/124.9.1720

[36] Nishijo H, Ono T, Uwano T, Kondoh T, Torii K. Hypothalamic and amygdalar neuronal responses to various tastant solutions during ingestive behavior in rats. The Journal of nutrition. 2000;130(4S Suppl):954s–9s. Epub 2000/03/29. 10736360 10.1093/jn/130.4.954S

