# Supplementary material for: Flavour-enhanced cortisol release during gum chewing
Source: PLoS One. 2017 Apr 5;12(4):e0173475. doi: 10.1371/journal.pone.0173475 (PMC5381771; doi:10.1371/journal.pone.0173475)
Supplement: S1 Fig — (PDF) [file pone.0173475.s001.pdf]

平成２４年９月１４日

倫理審査結果通知書

所　　属　　歯科口腔外科学
実施責任者　　主任教授　浦出　雅裕　　殿

兵庫医科大学
学長　中　西　憲　司

受付番号　第　1318　号

研究等課題名　味や香りの変化がガム咀嚼時のストレスに及ぼす影響

さきに申請のあった上記研究等課題について平成２４年９月４日の倫理委員会で審査し、下記のとおり判定しましたので、倫理委員会規程第８条第４項の規程に基づき、通知します。

判定	(承　認)　　条件付承認　　変更の勧告<br>不承認　　非該当
理由又は勧告	
