# Supplementary material for: Flavour-enhanced cortisol release during gum chewing
Source: PLoS One. 2017 Apr 5;12(4):e0173475. doi: 10.1371/journal.pone.0173475 (PMC5381771; doi:10.1371/journal.pone.0173475)
Supplement: S2 Fig — (PDF) [file pone.0173475.s002.pdf]

平成２４年９月１４日
September 14, Heisei24

倫理審査結果通知書
Ethic judgement results notification

所　　属　　歯科口腔外科学
Affiliation　oral science and maxillofacial surgery
実施責任者　　主任教授　浦出　雅裕　　殿
implementation supervisor　Chief Professor Prof. Masahiro urade

兵庫医科大学
Hyogo college of medicine
学長　中　西　憲　司
Vice-Chancellor Kenji Nakanishi
official seal

受付番号　第　1318　号
The reception desk number : 1318th

研究等課題名　味や香りの変化がガム咀嚼時のストレスに及ぼす影響
Research , etc. Subject The influence that the change of taste and the odor gives to stress by the gum-chewing

さきに申請のあった上記研究等課題について平成２４年９月４日の倫理委員会で審査し、下記のとおり判定しましたので、倫理委員会規程第８条第４項の規程に基づき、通知します。
We examine about the research theme in advance of the application mentioned above in an ethics committee of September 4, Heisei 24, we determined Based on official regulations of ethics committee official regulations Article 8 Clause 4,as follows. We notify you.

判定 Decision	(承認) Approval　不承認 Disapproval　条件付承認 Conditioning approval　非該当 Non-pertinence　変更の勧告 Advice of the change
理由又は勧告 A reason or advice	
