# Supplementary material for: Flavour-enhanced cortisol release during gum chewing
Source: PLoS One. 2017 Apr 5;12(4):e0173475. doi: 10.1371/journal.pone.0173475 (PMC5381771; doi:10.1371/journal.pone.0173475)
Supplement: S1 Text — (PDF) [file pone.0173475.s003.pdf]

※受付番号　第　　　　号

平成　　年　　月　　日

倫理審査申請書

兵庫医科大学長　殿

実施責任者	所属	歯科口腔外科学講座
	職名	主任教授
	氏名	浦出　雅裕

倫理委員会規定第8条の規定に基づき、下記のとおり申請します。

1．研 究 等 課 題

味や香りの変化がガム咀嚼時のストレスに及ぼす影響

2．実　施　期　間　　平成24年　9月　1日から　平成28年　3月31日まで

実　施　場　所

唾液の収集　兵庫医科大学　歯科口腔外科学講座

試料の準備，分析　兵庫医科大学　歯科口腔外科学講座　および　共同研究施設

3．研究等の概要

味や香りが様々な食品咀嚼した時のストレスを評価するために，ガム咀嚼中に分泌される唾液を採集しストレス関連物質である唾液中のコルチゾールを測定し，食品摂取時の感情とストレスとの関連性を検討する．

委員会開催年月日	平成　　年　　月　　日（　）			開催場所	
整理番号	第　　　　号	備　　考			

※				

（※印は記入しない。）

実 施 計 画 書

研究等課題	味や香りの変化がガム咀嚼時のストレスに及ぼす影響			
研究等組織	氏　　　名	所　属	職　名	担　当　の　分　野
実 施 責 任 者	浦出　雅裕	歯科口腔外科	主任教授	研究の総括
分 担 者	長谷川　陽子	歯科口腔外科	助教	使用装置の製作と調製
期　　間	平成 24 年　9 月　1 日から　平成 28 年　3 月 31 日まで			
場　　所	（〒663-8501）西宮市武庫川町1－1　兵庫医科大学病院　歯科口腔外科学			

１.研究等の実施目的：　何を、どこまで明らかにしようとしているのか具体的に記入のこと。

味や香りが様々な食品咀嚼した時のストレスを評価するために，ガム咀嚼中に分泌される唾液を採集しストレス関連物質である唾液中のコルチゾールを測定し，食品摂取時の感情とストレスとの関連性を検討する.その結果より，ストレスを惹起しやすい味/香り/味と香りの組み合わせを明らかにすることを目的にしている.

２.研究等の実施計画及び方法：　目的を達成するための計画及び方法を具体的に記入のこと。

概要

20～40歳の本学職員および学生のうち本研究の趣旨および安全性を説明し，実験参加への同意を得たボランティアの健常被験者を対象に，安静時唾液，および４つの味成分（酸味・甘味・苦味・塩味)と香り(レモン，無し)含んだガムを咀嚼した時に出る唾液を収集し，唾液に含まれるストレスマーカーとなるホルモン　(コルチゾール)をELISA法によって測定する．また，それぞれのガム咀嚼後に，味/香りについてのVisual analog scaleについて回答してもらい，主観的なストレスについても評価する.

1）対象

採用基準：

①健常有歯顎者で連続的にガム咀嚼が可能

②非喫煙者

除外基準：

本研究の趣旨および安全性を説明し，同意を得ることができない者

すべての実験は食後３時間以上あけて行い，コルチゾールが著しく高い早朝と低い夜間の実験は避ける．実験時の被験者の姿勢は座位とし，室温は２５℃に設定する．研究に使用するガムは，咀嚼１分間の咬筋筋活動に有意差が無いように，事前に同じ硬さになるよう調整する．

被験者は，兵庫医科大学の職員および学生の中から，20-40歳で研究参加の同意を得られた者を対象にした．目標は100名以上とし，男女比が同じになるようにリクルートする．

2)実験方法

実験で収集した唾液は，遠心分離(4000rpm，5分間)を行い，上澄み100μlをエッペンドルフチューブに分注し重量を計測した後，冷凍保存する．唾液中のコルチゾールの定量にはCortisol Parameter TM Assay Kit (R&D System, Minneapolis, MN)を用い，96wellマイクロプレートの吸光度計測にはUV/VISマイクロプレート分光光度計(SPECTRAmax PLUS384，モレキュラ・デバイス社製) をもちいる．

被験食品は，咀嚼開始から1分で同程度の硬さになるように調整した5種類のガム：①無味無臭ガム(gum base, 以下C-gum)，②味無し/レモンの香り(gum with odor, 以下O-gum)，③甘味料添加/香り無し (gum with taste, 以下T-gum)，④レモンの香り/甘味料を含む市販のガム(gum with taste and odor, 以下TO-gum, Free zone lemon ,Lotte,Tokyo, Japan)とする．すべてのガムはLotte社が製造し，ガムの硬さはTOの咀嚼開始1分後の硬さと同程度に事前に調整する．また，O-gum /SO-gum /TO-gumのレモンの香り，T-gum/TO-gumの味は同一とする．

計測は先ず，安静時唾液を1分間収集する．

次に，5種類のガムをそれぞれ1分間咀嚼する．咀嚼速度は自由とし，それぞれのガムを咀嚼中に分泌された唾液は50ml遠沈管(Iwaki社製, Tokyo, Japan)に収集する．被験食品は咀嚼直前に験者が被験者に渡し，咀嚼後は被験者自らが廃棄する．

咀嚼後，被験者はそれぞれのガム咀嚼後に，味/香りそれぞれについてのVisual analog scale(VAS;全くひどい味（香り）～素晴らしく美味しい（いい香り))に回答し，その後ミネラルウォーターで洗口する．すべてのガム咀嚼速度は被験者の自由とし，咀嚼と咀嚼の間には，３分以上空ける．

3) 中止条件

１．実験中に、対象者に有害事象が発生した場合。
２．対象者が参加を撤回した場合
３．験者が中止を必要と判断した場合

4) 研究参加者の費用負担

本研究に用いる実験機器や分析にかかわる費用は、験者らが一切を負担する

5) 補償

有害事象が発生した場合の金銭的補償は行わない。ただし、医療的に適切に速やかに対応する。

6) データの管理

本研究において得られたデータは、連結可能な匿名化をおこなったのち、電子メディアにて保存する。

ファイルにはパスワードをかけ、研究担当者以外には閲覧できないようにする。電子メディアは兵庫医科大学歯科口腔外科部門教授室のカギのかかる収納棚に保管する。また、データ処理に当たっては、外部接続のないコンピュータにておこなう。

Ⅲ. 研究等における医学倫理的・社会的配慮について

(1) 研究等の対象となる個人の人権擁護について

本研究は「臨床研究に関する倫理指針（平成１５年厚生労働省告示第 255 号、平成 20 年度全面改定)」に準拠しておこなう。

(2) 研究等の対象となる者に理解を求め同意を得る方法について

本研究を開始する前に、試験担当医師は以下の内容を含んだ説明書を説明し、被験者が説明内容を十分に理解したことを確認した上で、本研究への参加について本人の自由意思による同意を文書にて受領する。

イ　当該臨床研究への参加は任意であること

ロ　当該臨床研究への参加に同意しないことをもって不利益な対応を受けないこと

ハ　被験者又は代諾者等は、自らが与えたインフォームド・コンセントについて、いつでも不利益を受けることなく撤回することができること

ニ　被験者として選定された理由

ホ　当該臨床研究の意義、目的、方法及び期間

へ　研究者等の氏名及び職名

ト　予測される当該臨床研究の結果、当該臨床研究に参加することにより期待される利益及び起こりうる危険並びに必然的に伴う不快な状態、当該臨床研究終了後の対応

チ　被験者及び代諾者等の希望により、他の被験者の個人情報保護や当該臨床研究の独創性の確保に支障がない範囲内で、当該臨床研究計画及び当該臨床研究の方法についての資料を入手又は閲覧することができること

リ　被験者を特定できないようにした上で、当該臨床研究の成果が公表される可能性があること

ヌ　当該臨床研究に係る資金源

ル　当該臨床研究に伴う有害事象に対する金銭的補償はないこと

カ　問い合わせ、苦情等の窓口の連絡先等に関する情報

(3)　研究等によって生ずる個人への不利益並びに危険性に対する配慮について

①個人情報の漏洩によって、試料提供者のプライバシーを侵害する恐れがある。

①については，「個人情報の保護の方法」の項に記した対策を実行することによって発生を未然に防ぐことができる。

(4)　医学並びに社会への貢献度の予測について

咀嚼する食品の味や香りで摂取者のストレスが変化するのであれば，食事をおいしく・楽しく食べる生理的意義の一端を明らかにすることができ，同時に口腔機能の維持および回復の意義について，新たな見地からエビデンスを付加することができると予想される.

味や香りがガム咀嚼時のストレスに及ぼす影響を明らかにする試みにご協力ください.

安静時，およびガム咀嚼時に分泌される唾液を対象とした解析を受けていただくことについてのお願い

上記のお願いに際しまして，以下の12項目の説明をさせていただきます.説明をお聞きになられた上で，試料（唾液）提供にご協力いただけます場合，御自筆での同意書への御署名をお願いいたします.

ご協力の程，よろしくお願い申し上げます.

なお，この内容に関してご質問，ご意見などありましたら，歯科口腔外科の担当歯科医師までご遠慮なくお申し付けください.

1. **研究題目**

味や香りがガム咀嚼時のストレスに及ぼす影響

2. **研究期間の予定**

平成24年　9月　1日から　平成28年　3月31日まで

3. **研究の目的について**

食事中に生じる，「おいしい」という情動は，心を和ませ筋の緊張を解いて，精神的肉体的にリラックスさせる作用を持っており，咀嚼とこうした情動の関係を知ることは，咀嚼を単に消化の出発点だけでなくQOLを向上させ脳および全身の機能維持に貢献する日常的な運動としてとらえる上で重要です.また，食品の種類によって食事によるストレスが変化することは予想されていますが，詳細は未だ不明です.

本研究は，味や香りが様々な食品咀嚼した時のストレスを評価するために，ガム咀嚼中に分泌される唾液を採集しストレス関連物質である唾液中のコルチゾールを測定し，食品摂取時の感情とストレスとの関連性を明らかにすることを目的としています.

この研究によって，咀嚼する食品の味や香りで摂取者のストレスが変化するのであれば，食事をおいしく・楽しく食べる生理的意義の一端を明らかにすることができると予想されます.

4. **研究の方法について**

本研究は，顎関節の痛みがない，健常有歯顎者を対処にしています.

安静時，および約5種類のガム咀嚼時に分泌する唾液を収集し，ストレス関連物質である唾液中のコルチゾールを測定します.また，ガム咀嚼後には，味と香りについて美味しい/美味しくない，についてのアンケート(Visual analog scale)に回答いただきます.

本研究は，以下の流れに従って，遂行させていただきます.

（1）安静時唾液の収集

1分間の安静時唾液を収集します.

（2）ガム咀嚼時に分泌する唾液の収集

実験には約5種類のガム（無味，甘みのみ，香りのみ，甘み＋酸味，甘み＋香り）を用意しています.

それぞれのガムを１分間咀嚼し，咀嚼中に分泌する唾液を容器に収集します．咀嚼後は，味と香りについてのアンケートに答えていただきます．

（３）<u>採取した唾液の生化学的分析</u>

各被験者から採取した唾液を対象として，ストレスマーカーであるコルチゾール濃度を測定します．

5. <u>研究によって得られる学問上の利益</u>

本研究の結果，食事中の感情を惹起させる味/香りを明らかにすることができれば，食事をおいしく・楽しく食べる生理的意義の一端を明らかにすることができ，同時に口腔機能の維持および回復の意義について，新たな見地からエビデンスを付加することができると予想しています．

また本研究に参加することで検査を受ける本人が金銭的な利益を受けることはなく，危険も伴いませんが，検査に伴い苦痛や不快さが生じた場合は，いつでも研究中断に応じることができます．

6. <u>プライバシーの保護について</u>

分析の際の情報管理には細心の注意を払います．いただいた唾液は直ちに匿名化し，符号のみを付けます．したがって，分析を行っている研究者は，試料が誰のものか分からないようになります．

7. <u>提供された試料の検査結果の開示について</u>

原則的に研究に協力してくださった患者さんの検査結果は開示いたしませが，開示を希望される場合は，結果をお知らせいたします．

8. <u>研究結果の公表について</u>

専門の学会および学術雑誌において報告します．

研究成果を発表する際に個人情報にかかわるデータが公表されることはありません．

9. <u>本研究終了後の試料の取り扱いについて</u>

得られた唾液は，原則として本研究のために使用します．

しかし，可能な限り，以下に記載する内容について，同意いただきたく存じます．

近年における科学の発展の速さは目を見張るものがあります．その結果，本研究の終了時期において，本研究課題にかかわる様々な事実が明らかとなっている可能性があります．このことを受けて，研究さらには科学の発展にご協力いただく皆様の御好意を無駄にしないためにも，採取させていただいた唾液は無駄なく使用させていただければと思います．つきましては，本研究終了後も皆様からいただいた唾液を保管させていただければと存じます．唾液は匿名化した状態で取り扱いが行なわれ，唾液を使い切るまで保管いたします．また他の研究テーマで皆様からいただいた唾液を使用する場合，改めて研究計画書を兵庫医科大学倫理審査委員会に研究計画の申請を行ない，その妥当性について審議を受けた後に実施するようにいたします．

10. <u>費用について</u>

本研究では被験者に負担していただくことはありません．

11. 研究から生じる知的財産権の所属

研究の結果として知的財産権が生じる可能性があります．その権利は研究機関や研究者に属し，あなたには属しません．

12. 唾液提供を断ることについて

あなたが唾液提供を断ることによって，不利益を被ることはありません．

また，唾液提供後に同意を撤回することも可能です．その場合，説明担当者に御連絡いただければ，手続きの方法をお知らせし，採取した唾液および結果のすべてを廃棄し，得られたデータについても，一切，本研究に使用いたしません．ただし，同意を取り消された時点で，既に研究の成果が論文などによって既に公表された場合については，その限りにありません．

研究分担協力講座および担当者

兵庫医科大学医学部歯科口腔外科学講座　兵庫県西宮市武庫川町１－１，TEL:0798-45-6677　安川　陽子

同　意　書

兵庫医科大学長
病院長殿

この度、私は「研究課題　味や香りの変化がガム咀嚼時のストレスに及ぼす影響」（研究代表者　岸本裕充）に関する研究について、担当医師（＿＿＿＿＿＿＿＿＿＿）から、下記の項目につき、別紙の説明文書に基づき十分な説明を受け納得しましたので、研究に参加することに同意します（確認のため各項目にチェックしました）。

□　１）「研究の目的と意義及び方法と期間」
□　２）「研究対象者として選ばれた理由」
□　３）「研究への参加が任意であること」
□　４）「研究への参加に同意しなくても何ら不利益を受けることはないこと」
□　５）「研究への参加に同意した場合であっても随時これを撤回できること」
□　６）「研究に参加することで期待される利益及び起こりうる危険並びに必然的に伴なう不快な状態」
□　７）「この研究に係る資金源、研究者等の関連組織との関り」
□　８）「個人情報の取り扱い」
□　９）「研究計画書の開示」
□　10）「費用負担」
□　11）「補償について」

同意日　平成＿＿年＿＿月＿＿日

住所　〒＿＿＿＿＿＿＿＿＿＿＿＿＿＿＿＿＿＿＿＿

電話番号＿＿＿＿＿＿＿＿＿＿＿＿

本人氏名(自署)＿＿＿＿＿＿＿＿＿＿＿＿＿

説明日　平成　　年　　月　　日
所属　兵庫医科大学　歯科口腔外科学講座

説明者(自署)＿＿＿＿＿＿＿＿＿＿＿＿＿㊞

＊　この同意書は研究終了まで保管され、同意書のコピーは同意された本人にお渡しします。
＊　不明な点がありましたら、遠慮なく説明者にお尋ね下さい。

同　意　撤　回　書

兵庫医科大学
病院長殿

この度、私は「研究課題　干渉波刺激装置を用いた唾液分泌促進効果の比較検討試験」（研究代表者　岸本裕充）に関する研究に参加することに同意しましたことを撤回いたします。

平成______年______月______日

住所　〒____

本人氏名(自署)________________________________

同意撤回の意思を確認いたしました。

平成______年______月______日

所属　兵庫医科大学　歯科口腔外科学講座

署名______________________________

（＊研究者は同意撤回書の確認書のコピ-1 部を必ず受け取り保管してください）
