# Supplementary material for: Flavour-enhanced cortisol release during gum chewing
Source: PLoS One. 2017 Apr 5;12(4):e0173475. doi: 10.1371/journal.pone.0173475 (PMC5381771; doi:10.1371/journal.pone.0173475)
Supplement: S2 Text — (PDF) [file pone.0173475.s004.pdf]

※Receipt Number:
Date:

Application Form for the Ethical Review

President of Hyogo College of Medicine

Person in charge	Department	Department of Oral Science and Maxillofacial Surgery
	Job title	Chief professor
	Name	Masahiro Urade

In accordance with the provisions of Article 8 set forth by the Ethics Committee, I hereby apply for the ethical review as follows:

１. Running title
The influence that the change of taste and the odor gives to stress by the gum-chewing

２. Implementation period
From Heisei 24 Sep 1 to Heisei 28 March 31

3.Implementation site
Hyogo College of Medicine, Department of Oral Science and Maxillofacial Surgery of the Hospital of Hyogo College of Medicine

4.Brief overview of the study
To evaluate stress when taste and an odor performed various food chewing, we search for brain behavior functions during gum chewing and investigate psychological/ physical change at food intake and a relationship with the stress.

※

Date of the Ethics Committee		Place	
Serial number	No.	Note	

(※Mark can not fill)

Implementation plan document

<table>
<tr><td>Running title</td><td colspan="5">The influence that the change of taste and the odor gives to stress by the gum-chewing</td></tr>
<tr><td rowspan="3">Study Group</td><td colspan="2">Name</td><td>Position</td><td>Job title</td><td>A field of the charge</td></tr>
<tr><td>Person in charge</td><td>Masahiro Urade</td><td>Oral Science and Maxillofacial Surgery</td><td>Chief professor</td><td>Study design, implementation, presiding the study</td></tr>
<tr><td>Assigned person</td><td>Yoko Hasegawa</td><td>Oral Science and Maxillofacial Surgery</td><td>Associate professor</td><td>Data analysis, presiding the study</td></tr>
<tr><td>Period</td><td colspan="5">From Heisei 24 Sep 1 to Heisei 28 March 31</td></tr>
<tr><td>Place</td><td colspan="5">Hyogo College of Medicine, Department of Oral Science and Maxillofacial Surgery of the Hospital of Hyogo College of Medicine</td></tr>
</table>

1.The purpose of the study: Describe in detail the purpose of the study, its objective and the targeted result.

We collect saliva secreted during gum chewing and measure saliva cortisols which is a stress association material to evaluate a stress when a taste and odor performed various food chewing and examine the feeling at food uptake and the relationship with the stress.
As a result, it is intended that we determine a combination of taste / odor / taste and odor that are easy to cause a stress more.

2.The study plan and methods: Describe in detail the purpose of the study, its objective and the targeted result.

Profile

We explain a purpose of this study and safety among a 20-40-year-old staff of this school and students and, in the volunteer normal subjects who obtained consent to experiment participation, collect resting saliva and the saliva which appears when we chewed the gum which included four taste components (sour taste, sweet taste, bitter taste, salty taste) and odor (there is no lemon) and measure a stress marker included in the saliva and a hormone (cortisol) that it is by ELISA law. Also, after each gum chewing, we have you reply it about Visual analog scale about the taste / odor and evaluate the subjective stress.

1) **Subject**

Recruitment standards:

①Gum chewing is possible in normal

②Non-smokers

Exclusion criteria:

Describe the purpose and safety of the study, who is unable to obtain the consent

All experiments are over after a meal more than three hours and are carried out and avoid the early morning when a cortisol is remarkably high and the low night experiment. The position of the subjects at experiment assumes it sitting position, and the room temperature sets it to 25 degrees Celsius. We adjust the gum to use for research to the same hardness beforehand so that masseter muscle activity of chewing one minute does not have significant difference so that it is.

The subjects intended for a person obtained study entry's consent at 20-40 years old from a staff of Hyogo College of Medicine and a student. We do the goal more than 100 people and make recruiting so that sex ratio agrees.

2) Methods

After conducting centrifugal separation (4000rpm, five minutes), and dividing supernatant fluid 100μl in Eppendorf tube, and having measured a weight, we cryopreserve the saliva which we collected by an experiment. We use UV/VIS microplate spectrophotometer (SPECTRAmax PLUS384 leaks out and produces curadevice shrine) for the extinction mensuration of the 96well microplate to the determination of the cortisol in saliva using Cortisol Parameter TM Assay Kit (R & D System, Minneapolis, MN).

Five kinds of gum that we adjusted the test food to become the similar hardness from chewing start in one minute: (1) Do it with the commercial gum (TO-gum, Free zone lemon ,Lotte,Tokyo, Japan gum with taste and odor, or less) containing the odor / edulcorant of the ④ lemon which there are no gum (C-gum gum base, or less) without taste or odor, the odor (O-gum gum with odor, or less) of the / lemon which there is no ② taste in, ③ edulcorant addition / odor in (T-gum gum with taste, or less). Lotte company produces all gum and regulates the hardness of the gum to the same extent as hardness one minute after the chewing start of the TO beforehand. Also, it is assumed that the odor of the lemon of O-gum /SO-gum /TO-gum, the taste of T-gum/TO-gum are the same.

At first the mensuration collects resting saliva for one minute.

Then, we chew five kinds of gum for one minute, respectively. It is assumed that the chewing speed is free and collects 50 ml of saliva that each gum was secreted during chewing to a centrifuge tube (product made in Iwaki company, Tokyo, Japan). A person of omen hands the test food to subjects just before chewing, and subjects oneself scraps it after chewing.

The subjects reply Visual analog scale (VAS, totally terrible taste (odor) ... (good odor) delicious fantastically) about each taste / odor after each gum chewing and, after chewing, do mouth rinsing with mineral water subsequently. All gum chewing speed assumes it the freedom of subjects and, between chewing and the chewing, empty it more than three minutes.

3)Cancellation conditions

- When during an experiment, an adverse event occurred in subjects.
- When subjects withdraw participation
- When examiner determines that require experimentation stop

4)The costs burden on study participant

Costs associated with laboratory equipment and analysis to be used in the present study, the examiner pay for all.

5)Compensation

The financial compensation when an adverse event occurred is not carried out.
However, we cope immediately appropriately medically.

6) The management of data

After having conducted the coding that we can connect, we save the data obtained in this study in an electronic media. We bet a password on a file and prevent you from reading it other than the study person in charge. The electronic media keeps it on such a storing shelf of the key to professor at Hyogo College of Medicine dentistry and oral surgery section room. In data processing, it is carried out with a computer without the external connection.

Ⅲ. Medical ethics and social consideration in the study

(1) Protection of personal human rights of the subjects of the study

This study shall be conducted in compliance with Ethical Guidelines for Clinical Studies (Ministry of Health, Labor, and Welfare Bulletin No.255, Revision of FY 2008).

(2) Obtaining the consent from the subjects of the study

Prior to the commencement of the study, the physician in charge of the experiment shall explain to the subject the following information, and upon confirming that the subject fully understands, obtain a written agreement of the subject on participation by his/her free-will.

A: Participation in this clinical study is on a voluntary basis.

B: Refusal of participation in the study shall not result in an unfair treatment.

C: The subject or legal representative is entitled for revocation of his/her informed consent, without incurring unfair treatment at any given time.

D: Reason for selection as the subject

E: The signification, purpose, method and period of the study

F: The researchers names and job titles

G: The expected benefit from the participation of the study and the study result,
and possible dangers and the accompanying discomfort, post-experiment support

H: Upon the request by the subject or legal representative, he/she is entitled to obtain or browse information regarding the study implementation plan and methods, as long as doing so shall not violate the privacy information control of the other subjects, or the securement of originality of the clinical study.

I: The possibility of the disclosure of the study result, after anonymizing the data.

J: The funding source of this study

K: No monetary compensation shall be made for adverse event occurred to the subject during the experiment.

L: Contact information for inquiry and complaint window

(3) Consideration for personal disadvantage and risk caused by the study

1. By a leak of the personal information, we might infringe the privacy of the sample provider.

About 1, we can prevent occurrence by taking the measures that we wrote it down in the term of "6) The management of data".

(4) Expected contribution to medicine and the society

If the stress of the person of uptake changes with a taste and the odor of the food to chew, we can define a part of physiological significance to eat a meal happily deliciously. Simultaneously, about the significance of the maintenance of the oral cavity function and the recovery we can add evidence from the new standpoint.

Please cooperate with a conatus to clear the influence that a taste and an odor bring to a stress at gum chewing.

Invitation about having you receive the analysis for saliva secreted at a rest and gum chewing

On the occasion of the request mentioned above, we explain following 12 items. When you can cooperate with a sample (saliva) offer after explanation was heard, a signature to the consent form by the handwriting.
Please cooperate.

In addition, please order it to a charge dentist of oral surgery without reserve if there are a question, an opinion about these contents.

1. Research title
   The influence that the change of taste and the odor gives to stress by the gum-chewing

2. Plan of the study period
   From September 1, 2012 to March 31, 2016

3. About the purpose of the research
   It makes a heart soften, and the affect "to be delicious" to occur during a meal relaxes the muscle, and it has a psychological effect to relax physically, and it is important to know chewing and these emotional relation in arresting chewing as a daily motion to merely improve not only the starting point of the digestion but also QOL, and to contribute to a brain and generalized function maintenance. In addition, it is expected by the kind of the food that a dietary stress changes, but the details are still unknown.
   We collect saliva secreted during gum chewing, and this study measures saliva cortisol which is a stress-related substance to evaluate a stress when a taste and an odor performed various food chewing and is intended that we clear the feeling at food uptake and the relationship with the stress.
   If the stress of the person of uptake changes with a taste and the odor of the food to masticate, it is expected by this research when we can clear a part of the physiological significance to eat a diet happily deliciously.

4. About the method of the research
   This study makes a normal toothed jaw person without a temporomandibular pain a coping. We collect saliva to secrete at a rest and approximately five kinds of gum chewing and measure a cortisol in saliva which is a stress-related substance. In addition, after the gum chewing, please reply a

questionnaire about a taste and the odor.

This study accomplishes it according to the following flows.

(1) Collection of resting saliva
We collect one-minute resting saliva.

(2) Salivary collection to secrete at gum chewing
We prepare approximately five kinds of gum (it is tasteless, and, only as for the sweetness, only an odor is fragrant sweetness + acid taste, sweetness +) for an experiment. We masticate each gum for one minute and collect saliva to secrete during chewing to a cell.
After chewing, please answer the questionnaire about a taste and the odor.

(3) The salivary biochemical analysis that we obtained
In saliva which we obtained from each subject, we measure the cortisol concentrations that are a stress marker.

5. Benefit in the study to be obtained by a research
We can clear one end of the physiological significance to eat a diet happily deliciously if we can determine taste / odor to make a feeling at table caused as a result of this study. About the significance of the maintenance of the oral cavity function and the recovery, we expect that we can add an evidence from the new standpoint at the same time.
In addition, it is not associated with the risk to call that oneself who is examined by participating in this study receives financial benefit either, but can accept a research intermission anytime when pain and jar occur with an examination.

6. About protection of the privacy
We pay scrupulous attention to the documentation in case of the analysis.
Saliva which we had codes you promptly and gives only a sign.
Therefore, a sample is whose thing, or the researcher analyzing it does not come to be found.

7. About the disclosure of the test result of the offered sample
The test result of the patients who cooperated in principle with research does not disclose it, but will tell you about results when disclosure is hoped for.

8. About the publication of results
We report it in a specialized society and a learned journal.
When we present the results of research, data about personal information may not be published.

9. About the handling of the sample after this study

As a general rule, we use obtained saliva for this study.

However, please agree on a content to describe below as much as possible.

The speed of the scientific development in late years has a thing to stare wide-eyed at.

As a result, in end time of this study, various facts about this study problem may be found.

Receive this, and a research less wastes light of your countenance of sciences having a progress cooperate; feel to accumulate it that can make work saliva which obtained without waste.

Therefore, we think that you can make saliva which all of you gave to me after this study kept.

The handling is carried out with the state that we coded, and saliva keeps it until we use up saliva.

In addition, we apply to the Hyogo College of Medicine Ethical Review Board for the research design and perform research program documentation some other time when we use saliva which all of you gave to me on other research themes after having received the deliberation about the validity.

10. About costs

We may not have subjects bear it in this study.

11. Position of the intellectual property rights that a research causes

Intellectual property rights may occur as a result of research.

The right is vested in a research institute and a researcher and is not vested in you.

12. About rejecting a saliva offer

We may not take a disadvantage by your rejecting a saliva offer.

In addition, we can withdraw an agreement after a saliva offer.

In that case, if the explanation person in charge can inform me, we tell you about the method of the procedure and discard all of saliva which we obtained and results and do not use it in this study about the obtained data at all either.

However, there are none in the limit when we are accompanied when an agreement was canceled when the result of the research is already announced by papers.

A research allotment cooperation lecture and the person in charge

Hyogo College of Medicine, Department of Oral Science and Maxillofacial Surgery, Hyogo College of Medicine, 1-1 Mukogawacho, Nishinomiya, Hyogo, 663-8501

TEL: 0798-45-6677 Yasukawa proton

Consent form

President of Hyogo College of Medicine
Director of a hospital

This time I am doctor attending about a study on "effect that a change of a research theme taste and the odor gives to a stress at gum chewing" (research representative Hiromitsu Kisihimoto) Because we understood it in response to enough explanation based on the explanation document of the attached sheet from) in the following item, we agree to participating in research (we checked it for an identification in each item).

□ 1) "A purpose and significance of the research and a method and a period"
□ 2) "The reason that was chosen as study patients"
□ 3) "Participation in research being arbitrary"
□ 4) "Not achieving any disadvantage even if we do not agree to participation in research"
□ 5) What "can withdraw this at any time even if we agree to participation in research"
□ 6) "Benefit prospective in participating in a study and a possible risk and an unpleasant state to be associated with necessarily"
□ 7) "A relation with the related histology such as a source of funds, the researcher concerned with this study"
□ 8) "A privacy policy"
□ 9) "The disclosure of the research plan"
□ 10) Costs burden"
□ 11) About "a compensation"

Agreement day Heisei age month day

Address 〒______________________________

Phone number______________________________

Person full name (signature)______________________________

Explanation day Heisei age month day
Regional Hyogo College of Medicine dentistry oral surgery lecture

Explainer (signature)______________________________ ㊞

＊ This written consent is kept until the research end and hands the copy of the consent form to the consensual person.
＊ If you have any questions, ask an explainer without reserve.

Consent withdrawal statement

Hyogo College of Medicine

Director of a hospital

This time I withdraw that I agreed that I participate in a study on "comparison study of the salivation acceleration effect using the research theme interferential currents stimulator" (research representative Hiromitsu Kishimoto).

Agreement day Heisei age month day

Address 〒____

Phone number______________________________________

Person full name (signature)____________________________

We confirmed a bulesis of the agreement withdrawal.

Heisei age month day

Regional Hyogo College of Medicine dentistry oral surgery lecture

Signature______________________________________

(you receive -1 copy of copy of the confirmation of the agreement withdrawal book by all means, and please keep □ researcher)
